# Audiovisual Perception of Sentence Stress in Cochlear Implant Recipients

**DOI:** 10.3390/audiolres15040077

**Published:** 2025-06-24

**Authors:** Hartmut Meister, Moritz Wächtler, Pascale Sandmann, Ruth Lang-Roth, Khaled H. A. Abdel-Latif

**Affiliations:** 1 Department of Otorhinolaryngology, Head and Neck Surgery, University Hospital of Cologne, Kerpenerstr. 62, 50937 Cologne, Germany; 2 Jean Uhrmacher Institute, University of Cologne, Geibelstraße 29-31, 50931 Cologne, Germany; 3 Department of Otolaryngology, Head and Neck Surgery, Carl-von-Ossietzky University of Oldenburg, Steinweg 13-17, 26122 Oldenburg, Germany; 4 Cluster of Excellence ‘Hearing4all’, University of Oldenburg, 26129 Oldenburg, Germany

**Keywords:** cochlear implants, sentence stress, prosody, reaction times, pupil dilation, eye gaze, virtual reality

## Abstract

**Background/Objectives:** Sentence stress as part of linguistic prosody plays an important role for verbal communication. It emphasizes particularly important words in a phrase and is reflected by acoustic cues such as the voice fundamental frequency. However, visual cues, especially facial movements, are also important for sentence stress perception. Since cochlear implant (CI) recipients are limited in their use of acoustic prosody cues, the question arises as to what extent they are able to exploit visual features. **Methods:** Virtual characters were used to provide highly realistic but controllable stimuli for investigating sentence stress in groups of experienced CI recipients and typical-hearing (TH) peers. In addition to the proportion of correctly identified stressed words, task load was assessed via reaction times (RTs) and task-evoked pupil dilation (TEPD), and visual attention was estimated via eye tracking. Experiment 1 considered congruent combinations of auditory and visual cues, while Experiment 2 presented incongruent stimuli. **Results:** In Experiment 1, CI users and TH participants performed similarly in the congruent audiovisual condition, while the former were better at using visual cues. RTs were generally faster in the AV condition, whereas TEPD revealed a more detailed picture, with TH subjects showing greater pupil dilation in the visual condition. The incongruent stimuli in Experiment 2 showed that modality use varied individually among CI recipients, while TH participants relied primarily on auditory cues. **Conclusions:** Visual cues are generally useful for perceiving sentence stress. As a group, CI users are better at using facial cues than their TH peers. However, CI users show individual differences in the reliability of the various cues.

## 1. Introduction

For several decades, cochlear implants (CIs) have been the treatment of choice for the rehabilitation of severe-to-profound hearing loss when hearing aids do not provide sufficient benefit. CIs aim to restore hearing through electrical stimulation of the auditory nerve and in many cases give the user the opportunity to understand speech, at least in acoustically favorable environments. Despite the great success in supporting verbal communication, CIs show areas of possible improvement, especially due to the fact that electrical stimulation based on a restricted number of electrodes limits the transmission of spectro-temporal cues. This is evident, for example, in the discrimination and identification of voices (overview in [1]) or in the perception of affective or linguistic prosody (overview in [2]).

Prosody provides valuable information that goes beyond syntax or semantics. Affective prosody refers to emotions, such as happiness, sadness, anger, and fear, and thus gives information about the talker’s mood. Linguistic prosody reflects the talker’s intention and the meaning of an utterance, for example, by characterizing it as a declarative or interrogative sentence or by highlighting important words within a phrase. The latter is known as sentence stress and refers to the degree of prominence of individual words, which can be used to emphasize new information in an utterance. A contrastive accent, for example, is used to put the focus on a particularly significant word in a sentence, such as in “The BOY wears a red pullover, not the girl”.

Various acoustic cues like syllable duration, intensity, and the fundamental frequency of the voice (F0) play a role in sentence stress. Stressed syllables usually exhibit a longer duration, a higher intensity, and a higher fundamental frequency, although this can be specific to different languages. These features are typically readily available to listeners with healthy hearing. However, cochlear implant users may be significantly limited in this respect. Meister et al. [3,4] compared CI users and normal-hearing listeners in terms of their ability to discriminate these cues and to identify stressed words in natural utterances. The study used controlled manipulations of these features to obtain detailed information on the perceptual abilities of the participants. The results revealed that CI users showed significantly poorer discrimination for F0 and intensity compared to NH listeners, but not for syllable duration. The difference limens for F0 were about four- to five-times higher and for intensity about two-times higher than for the NH listeners, generally confirming the results of an earlier study [5]. Similarly, the identification of stressed words based on F0 and intensity was poorer, reflected in shallower psychometric functions, while duration resulted in similar identification as for the NH listeners. Finally, F0 and intensity were shown to contribute the most to the identification of stress in natural utterances, giving evidence for limitations in prosody perception in everyday life when using cochlear implants. These previous studies thus added to the literature showing limitations in spectro-temporal processing with CI and especially the importance of F0 as a suprasegmental speech feature.

However, it is not only acoustic features that play a role. A number of studies suggest that visual cues might also be important for prosody perception in general and sentence stress identification in particular (overview in [6]). For example, Kim et al. [7] recorded facial expressions (i.e., raising eyebrows) and head movements of a talker while he uttered a sentence with stress on a particular word (“use the PENCIL to write the first draught”). The authors found that the stressed word was preceded and accompanied by distinct eyebrow movements and changes in head position. Specifically, eyebrows were raised and the head was rotated in the vertical plane, both resulting in a characteristic visual pattern that can be used for stress identification, although the movements may vary between individuals [8,9].

A number of studies have shown that eyebrow raises and changes in F0 in particular are closely connected. For example, Guaïtella et al. [10] synchronously assessed voice production and eyebrow movements and found that eyebrow raising was associated with turn-taking and modulations in F0. Specifically, when F0 was increased, a corresponding movement of the eyebrows could be detected in the same temporal window (but see [11], who did not find such an alignment). Flecha-García [12] also examined temporal aspects of stressed words in various dialogues with regard to acoustic and facial cues. They found a close alignment between eyebrow movements and pitch accents, with eyebrow raises preceding the corresponding changes in F0 by 0.06 s on average. In another study, participants were instructed to read sentences aloud while holding their eyebrows in a lowered, neutral, or raised position [13]. The latter was found to be associated with a slight but significant increase in F0.

While the specific origin of the relationship between changes in F0 and eyebrow movements is still a matter of debate, it is likely that the interrelation represents a linguistic function that provides complementary or supplementary information compared to that in a single modality (overview in [8]). This raises the question of whether and to what extent CI recipients are able to use facial cues, such as eyebrow raises, to overcome limitations in the perception of acoustic prosody cues, such as F0. To our knowledge, only a few studies have addressed this question so far, mostly in the framework of affective prosody.

For example, Ambert-Dahan et al. [14] found that participants with hearing loss showed lower emotion recognition compared to their normal hearing peers, especially in an implanted study group. This finding was unexpected, as CI users typically have good visual abilities with regard to speech stimuli (e.g., [15,16,17]).

Fengler et al. [18] examined happy, sad, and angry expressions for unimodal, audiovisually congruent, and incongruent stimuli. The CI users performed auditorily worse than their NH counterparts and were more affected by incongruent visual stimuli. In contrast, no significant differences were found between the CI and NH participants in the visual modality.

Another study examined multimodal emotion recognition of anger and surprise [19]. As expected, CI recipients generally performed worse than age-matched NH participants in an auditory-only condition, but the former showed a larger benefit when congruent facial cues were added.

Recently, Yu et al. [20] compared Mandarin-speaking children with CIs and a group of normal-hearing peers regarding irony comprehension. Three conditions were considered, one with neutral utterances where contextual information could provide cues to the presence of irony, another with additional acoustic prosodic cues (i.e., corresponding changes in F0, intensity, and duration), and a third where additional facial information was given. While the CI children consistently showed a 20–25% lower irony comprehension than the NH children, both study groups benefited similarly from acoustic prosodic cues and additionally from visual cues.

With regard to *linguistic* prosody, an early study [21] tested prosodic contrasts in vowel length, word stress, and sentence stress in an auditory-only, a visual-only, and a congruent audiovisual condition. Groups of CI recipients and hearing-aid users were considered. The former showed a correct identification of prosodic contrasts of 75.3% in the visual-only, 81.8% in the auditory-only, and 85.2% in the congruent audiovisual condition and thereby generally performed about 10% worse than the hearing-aid users, particularly in the auditory-only condition, while visual cues were useful to a similar extent in both study groups.

Basirat [22] described a case study in which a group of normal-hearing subjects were compared with a post-lingually deafened CI recipient. Phonemically identical but prosodically different pairs, which differed mainly in the F0 contour, were presented in an auditory-only and audiovisual condition. While the NH subjects revealed a correct identification of about 80% in both conditions, the CI user showed only 62.1% in the auditory-only and 55.2% in the audiovisual condition—despite the fact that she was classified as an “exceptionally good” performer.

Lasfargues-Delannoy et al. [23] investigated the ability to distinguish between a question and a statement in CI users and normal-hearing controls. To this end, two actors presented different utterances as a declarative or interrogative sentence. To avoid ceiling effects, they were instructed to deliberately reduce prosodic features in both the visual and the auditory modality. Moreover, to avoid ceiling effects in the auditory modality in the NH participants, the utterances were subjected to an eight-channel vocoder. While this resulted in a similar group performance in the auditory-only condition, it was unexpectedly found that the control group was better at discriminating questions from statements than the CI recipients in the visual-only condition. However, when the two modalities were congruently combined, the CI users showed greater multi-sensory benefit, possibly indicating better integration of the modalities for question/statement identification.

Regarding the combination and integration of different modalities, several theoretical frameworks exist, such as the “Modality Appropriateness Theory” [24], the “Reliability-weighted Cue Integration” [25], or the “Maximum Likelihood Estimate” [26]. What these considerations have in common is that they assume that the modality-specific sensory signal quality can vary and that the corresponding information is weighted according to its reliability. Based on this weighted information, the modalities are combined. In the case of discrepancies, it is assumed that the more reliable modality dominates. Since CI users receive an underspecified auditory signal and have been described as having enhanced visual abilities in terms of speechreading [15,16,17], these mechanisms can lead to differing results compared to individuals with healthy hearing, who typically have access to more reliable auditory information.

Since the results of the studies presented above are inconclusive, the aim of the present investigation is to shed more light on the audiovisual perception of linguistic prosody in CI recipients. Specifically, the ability of experienced adult CI users to perceive sentence stress is examined in comparison to typical-hearing (TH) individuals of a similar age. A multimodal virtual-reality based speech presentation is applied to enable highly controlled manipulation of the underlying cues while retaining a high degree of life-like conditions, allowing a more robust method than has been previously used. In this case, the fundamental frequency as an important but potentially unreliable cue and eyebrow movements were manipulated to examine the participants’ perceptual abilities and the underlying mechanisms in more detail. Moreover, it follows a multidimensional approach in data collection in order to obtain more information about the mechanisms involved than has been captured before in a single study. Therefore, in addition to the proportion of correct stress perception, the load associated with accomplishing the task of identifying the stressed word was assessed using a behavioral measure (reaction times) as well as a physiological measure (task-evoked pupil dilation, TEPD). Finally, gaze fixation as an indication of visual attention (e.g., [27]) was also taken into account. Experiment 1 focuses on the use of each modality alone and their congruent combination. For comparability and in line with Lasfargues-Delannoy et al. [23], we balanced the performance of both groups in the auditory modality. Experiment 2 additionally considers incongruent combinations of the two modalities to detect a possible individual dominance in the use of auditory and visual cues.

Due to the rather sparse literature and partly contradictory results of the previous studies, it was difficult to formulate specific hypotheses. However, given the potentially high ability of CI recipients to use visual speech and considering the theories on the reliability-weighted use of cues in the different modalities, we expected them to show better stress identification in the visual-only mode than the TH control group. By contrast, based on the methodological design mentioned above, we did not expect a significant difference in the auditory-only condition. We further assumed that both study groups would perform better in the audiovisual condition than in the unimodal conditions. Still, following reports of better integration abilities [15,23], we anticipated that the CI users would benefit more from congruent audiovisual cues than the control group.

In terms of task load, we expected that the CI users would show higher reaction times and larger pupil dilation in the auditory-only than in the visual-only condition, and vice versa for the TH participants, as motivated by the theoretical framework of the reliability-weighted use of cues. Based on this framework, we anticipated that a successful combination of the cues in the single modalities would reduce task load in the congruent audiovisual condition, which should be particularly pronounced in the CI users. Conversely, following reports that combining auditory and visual information may be associated with an increased processing load [28,29,30], an alternative hypothesis would predict higher reaction times and pupil dilation for the congruent modality combination. Regarding visual attention as reflected by the eye gaze data, we expected both groups to primarily focus on the eye-region of the virtual talkers in order to assess the corresponding facial movements, i.e., eyebrow raises.

Regarding the incongruent stimuli in Experiment 2, we expected that the typical-hearing control group would predominantly rely on the auditory modality whereas CI users might predominantly use facial cues, giving more insight into the individual reliability-weightings of the two modalities.

## 2. Materials and Methods

### 2.1. Stimuli

The aim of the present study was to determine how controlled changes in F0 and facial movements influence the identification of lexical stress in a sentence and how perception in the single modalities combines in a congruent or incongruent audiovisual condition. To this end, the German sentence “Der Junge trägt einen roten Pullover” (“The boy wears a red pullover”) was used, which is part of a prosody test battery [31]. The stressed word either could be the subject (“Junge”), adjective (“roten”), or object (“Pullover”). The decision to use only one single sentence followed Swerts & Krahmer [32] to ensure that the participants focused exclusively on the prosodic cues under investigation and not on any lexico-syntactic variation. Moreover, this simplified the implementation of incongruent audiovisual stimuli, as applied in Experiment 2.

The stimuli were based on natural utterances of the sentence by a female (age 30 years) and a male talker (age 33 years), which were recorded at a sampling rate of 44.1 kHz using a SM 2 microphone (Sontronics^TM^, Poole, UK) routed to a Fireface soundcard (RME^TM^, Haimhausen, Germany) The two talkers had a mean F0 of 160 Hz and 108 Hz, respectively. Both the stressed versions and an unstressed neutral version of the sentence as a reference were recorded. Since the focus was on inducing stress by F0 changes, the talkers were instructed to keep the word timing and length of the utterances similar. Furthermore, any potential intensity differences between the stressed and neutral utterances, which could also indicate prominence, were removed using Adobe^TM^ Audition.

The core of the methods was a controlled modification in the corresponding cues to obtain detailed information about the participants’ perceptual abilities. Using a script based on the pitch-synchronous overlap-add (PSOLA) algorithm in *praat* [33], the F0 of the stressed syllable of the corresponding word was modified in different steps to finally approximate the contour of the neutral utterance, resulting in different modification levels. Such a “morphing” approach ensured that the intonation contour was generally preserved, resulting in much more natural-sounding stimuli than considering only F0 height but not shape [34].

Subsequently, pilot experiments were conducted to ensure that the neutral stimulus did indeed not contain any lexical stress information and that the modifications of F0 influenced the perception of prominence on the corresponding word as intended. Based on these pilot experiments, the range of F0 modifications was determined for both groups of participants, with the goal of avoiding both floor and ceiling effects. The aim was to present three different modification levels in order to cover a range of about 50% to 80% correct stress identification. Ultimately, compared to the neutral utterance, this resulted in increases of 10, 20, and 40 Hz in the stressed syllable for the group of CI users and 6, 8, and 10 Hz for the TH participants, who showed a clearly steeper psychometric function for F0 changes (see [4]). These values correspond to the increase in F0 relative to the neutral utterance, expressed as the average across the respective syllable (see Table 1).

**Figure 1:**
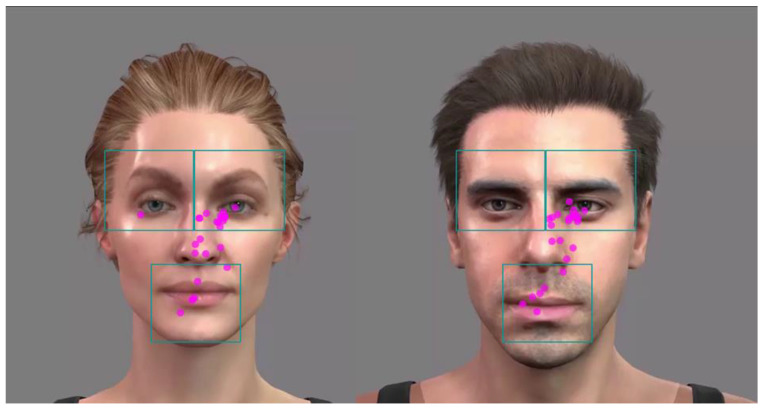
Female and male virtual character used in the experiments. The green squares show the areas of interest defined for the left and right eye and the mouth as used for the eye-tracking analysis. Pink dots reveal an example of gaze fixation during a stimulus interval.

While the approach of modifying the F0 of the acoustic signal is well established and widely used in language and hearing research for various purposes (e.g., [3,34,35,36,37]), modifying the visual cues is much more complex and not readily available. One option is to use video recordings of actors who are instructed to decrease or increase their facial movements while uttering the stimuli [23]. However, controlled manipulation of the underlying cues that targets predefined modification levels does not seem to be possible. In this regard, the use of virtual characters is a more promising approach because virtual reality offers the opportunity to present highly realistic scenes in a flexible manner. More specifically, it is possible to control virtual characters based on natural head and face movements and to arbitrarily modify the strength of the movements, which allows for a highly scrutinized stimulus setup. Here, we used two different virtual characters provided by the iClone v. 7. Package (Reallusion^TM^ Inc., Atlanta, GA, USA). These characters are shown in Figure 1 and represent the female and the male talker.

The talkers uttered the sentence in a simulated dialogue situation to create natural facial and head movements, which would not necessarily be possible if the sentence were simply read aloud [38]. In this dialogue situation, the motions of the two talkers were captured by an iPhone 13 (Apple Inc. Cupertino, CA, USA) running the “live face” app (Reallusion^TM^ Inc., Atlanta, GA, USA). This allowed the extraction of landmarks that provided the information for controlling the virtual characters (see [39] for details on this method). Based on this motion capture data, the characters can be animated, with the magnitude of the movements (“expression strength”) varying between 0% (static face) and 200% (doubling natural movements). A technical validation of the method demonstrated that it provides very similar head and eyebrow movements to video recordings and shows that the approach is generally suited to presenting highly realistic but parametrizable visual prosody cues [39]. Based on this technical validation and behavioral pilot data, we used three different “expression strengths” of 50, 30, and 10% of the natural movements (see Table 1). The rationale behind decreasing the head and facial movements compared to the natural expression (100%) of the real talkers was again to estimate the underlying psychometric function while avoiding floor or ceiling effects in the single modalities. Though we were particularly interested in eyebrow movements, since these play a major role for stress identification [32], we also took the captured head movements into account in order to provide stimuli that were as true to life as possible.

In addition to head and facial movements, the virtual characters also provided articulatory movements of the mouth. This was feasible by the “Accu Lips” module included in the iClone v. 7. Package. This module uses the written text of the sentence and an annotation of each syllable to produce realistic movements of the yaws, the lips, and the tongue. Articulatory movements were not necessarily required for the study objective, but were considered to create the most realistic impression of the virtual characters.

Based on the manipulations described above, Experiment 1 included auditory-only modifications of F0, visual-only modifications of the facial and head movements, and a congruent audiovisual condition by pairing the cues on each of the three modification levels (see Table 1). Importantly, the stimuli were always presented to the participants in an audiovisual manner: In the auditory-only modifications, facial and head movements were eliminated (i.e., “expression strength” = 0%) but the articulatory movements of the mouth were retained. In the visual-only manipulations, facial movements (as well as head and articulatory movements) were provided, but the neutral utterance of the sentence was always given as the time-aligned audio signal.

In order to determine whether individual participants predominantly used one of the two single modalities for perceiving sentence stress, Experiment 2 considered both congruent and *incongruent* combinations of the auditory and visual cues. The term “incongruent” reflects the fact that the stress in one modality does not match that in the other modality (e.g., F0 indicates stress on the subject, facial movements on the object). Following Swerts & Krahmer [32], in normal-hearing participants such a modality-specific dominance was particularly evident when stress was on the first or the last word of the sentence. Therefore, in contrast to Experiment 1, the stress was either only on the subject (“Junge”) or the object (“Pullover”) of the sentence. For this, we used the natural stressed utterances of the sentence, which had an average F0 increase of 45 Hz compared to the neutral utterance, as well as an “expression strength” of 100%, reflecting the natural facial and head movements. Thus, the F0 increase was slightly higher than the most pronounced modification level 3 of the CI recipients. To investigate whether possible modality-specific dominance changes when the underlying cues are less salient, we also used the stimuli based on modification level 2 in a congruent and incongruent manner.

### 2.2. Setting

The experiment was conducted in a dimly lit, sound-treated booth (2 m width, 3 m length, 2.4 m height). The virtual characters were displayed on a 23.8 inch flat-screen monitor (E243i, Hewlett-Packard^TM^, Palo Alto, CA, USA) at a distance of about 80 cm from the participant’s eyes. The time-aligned sentences were presented through a Rokit KRK5 free-field speaker (KRK Systems^TM^, Nashville, TN, USA), situated slightly above the monitor at a distance of 100 cm from the participant’s head and calibrated to a level of 65 dB SPL. Gaze and pupil size were determined via an SR Eyelink 1000 plus system (SR Research^TM^, Mississauga, Ottawa, Canada). For this purpose, the subject’s head was fixated using a chin rest after being instructed to find a stable but comfortable position. Lighting was mainly determined by the brightness of the monitor. Since pupil size is critically dependent on illumination, the brightness was set to an intermediate value that should provide sufficient headroom for changes in pupil responses. This value was determined based on the experiences of a pilot study and corresponded to 30 lux, a value that is recommended when testing older listeners [40], as is the case in the present study. Gaze and pupil size were recorded from the right eye at a sampling frequency of 500 Hz using a 25 mm lens (Eyelink High speed for DM-890/AM-890, SR Research^TM^, Mississauga, Ottawa, Canada). Gaze fixations were extracted from the Experiment Viewer software (SR Research Ltd., version 3.1, Mississauga, Ottawa, Canada) using the time course (binning) approach with a 20-millisecond binning interval. The exported data represented the “dwell time”, i.e., the percentage fixation time that falls within the predefined areas of interest (AoIs), which covered the left and the right eye as well as the mouth of the virtual characters (see Figure 1).

### 2.3. Stimulus Presentation

The virtual characters were presented as videos (mp4, 24 frames/s) on a flat-screen monitor. The height of the head was 22 cm for the female character and 23.5 cm for the male character, and the width was 15 cm in both cases, which corresponds to the actual size of a human head and yielded visual angles of 10.7° (horizontal) to about 16° (vertical). According to Hall [41], this relatively small visual angle and close distance correspond to the category of the “personal zone” of communication and ensured that the facial movements were well perceivable.

The videos started with a frozen image of the first frame for a duration of 2 s. This allowed the pupil to get accustomed to the presentation and enabled us to calculate a suitable baseline, which was chosen as the interval between 500 and 0 ms prior to the onset of the sentence. The sentence duration was approximately 1.7 s. The animated characters and the recording of the sentence were presented as time-aligned, and the last frame of the animation was displayed as a frozen image until the end of the trial, which terminated 8 s after the sentence start. This period was chosen as a post-stimulus phase in order to allow the pupil size to return back to its initial value before the next stimulus was presented.

To avoid sequence and habituation effects regarding the three different modality conditions, the stimuli were presented in mixed blocks. The mixed-block presentation makes it more difficult for the participants to learn or adapt to specific patterns on a trial-by-trial basis, which could promote the use of modality-specific strategies. Hence, the focus was more on a spontaneous and intuitive perception of sentence stress. Also of relevance, as outlined by Mechtenberg et al. [42], a mixed-block presentation is advantageous in terms of potential issues with anticipatory effects and the impact on pre-stimulus pupil dilation and baseline pupil size drift, which could confound the comparison of the different modalities.

In Experiment 1, each mixed block considered the 3 different modification levels (1–3), 3 different foci (subject, adjective, object), 3 different modalities (A-only, V-only, congruent AV) and the 2 virtual characters in a pseudo-random order. To cover all possible combinations, four different blocks of 27 stimuli each were presented, with each stimulus presented twice (but not within the same block). This resulted in a total presentation of 108 stimuli.

To investigate a possible modality-specific dominance in Experiment 2, two mixed blocks were presented. These contained both incongruent and congruent stimuli in a pseudo-randomized order (i.e., 2 virtual characters x 2 levels (modification level 2, natural) x 2 foci (subject, object) x 2 congruency conditions (congruent, incongruent)). To increase the stability of the data, each stimulus was presented four times, resulting in a total presentation of 64 stimuli (32 congruent, 32 incongruent).

### 2.4. Task and Procedure

The task of the participants was to indicate whether the stressed word was the subject, the adjective, or the object of the sentence (only the subject and the object in Experiment 2). For this purpose, these options were assigned to the left (subject), down (adjective), and right (object) arrow keys of a standard computer keyboard and labeled accordingly. Participants were instructed to place the index, middle, and ring finger of the right hand on the corresponding key and indicate the stressed word as accurately and as quickly as possible, yielding both performance and reaction time data. Since the response speed for the three words may vary due to motor differences, the reaction times for the three fingers were determined individually in advance by applying a simple visual 3-alternative forced choice paradigm, and used for correction by subtracting the manual RT from that obtained during the actual experiment.

Participants were advised not to sacrifice accuracy for speed and not to press any key if they felt unable to indicate the stressed word. The reason for the latter was that potentially conflicting information was given in Experiment 2 and we aimed to keep the task identical across both experiments and avoid guessing. Responses could be given at any time during the trial, that is, even before the end of the sentence. This scheme was used because it would not have yielded sensitive RT data if participants were only allowed to respond after sentence offset. With regard to task-evoked pupil dilation (TEPD), which was taken in the present study as a physiological indicator of the task load, this means that the interval of sentence presentation was not necessarily isolated from the participant’s response phase. Thus, it is not possible to readily distinguish between a stimulus-evoked pupil dilation and a response-evoked pupil dilatation, which is usually accounted for by including a retention interval (for a discussion on simultaneous measures of response time and pupil dilation, see [43]).

Experiment 1 began with collecting the informed consent of the participants and recording basic data such as gender, age, and type of and experience with the hearing device. Next, a familiarization phase was conducted by presenting 20 animations of the virtual characters, followed by an explanation of the task and practice list based on 18 animations that used the natural utterances of the sentences and the natural movements as well as the stimuli of modification level 3. This was repeated until it was clear that the participant had understood the task. After that, the participant’s head was positioned using the chin rest and the eye gaze was calibrated using the “Experiment Builder” of the SR Eyelink 1000 system. The gaze was assessed employing a nine-point calibration and validation procedure for each participant. This procedure was carried out before each block of trials. Then, two blocks of 27 stimuli each were presented in succession. This was followed by a break of about 10 min, after which the two remaining stimulus blocks were presented. After another break of about 10 min, Experiment 2 was conducted by presenting the two blocks of 32 stimuli each. In general, breaks were also possible after each of the blocks upon individual request. The total duration of the experimental session was about 90 min.

### 2.5. Participants

The main focus of the study was the comparison of sentence stress identification in the three modality conditions (A, V, AV) in CI recipients and typical-hearing peers. Considerations regarding sample size were based on differences between the AV and the unimodal conditions as well as interactions with the study group. Regarding the former, sample size calculations via G*Power ver. 3.1.9.7 [44] assuming a medium-sized effect (d’ = 0.25), an alpha-level of 0.05, and a power of 0.80 using pilot data of 5 CI recipients revealed a minimum sample size of 8 subjects. In terms of an interaction between the two study groups and the three modality conditions, a minimum of 14 subjects per group was calculated.

In total, 28 participants participated in the study: 14 experienced CI users who communicated in the oral mode and 14 typical-hearing (TH) participants. Implant experience was between 2 and 23 years (8.9 ± 5.9 yrs.) and hearing-aid experience between 4 and 64 years (34.4 ± 18.4 yrs.). One subject (CI04) was pre-lingually deafened and another five (CI02, CI03, CI12, CI13, CI14) reported hearing problems during childhood. Six participants were bilaterally fitted, one participant used one CI without residual hearing on the contralateral side, and seven used bimodal stimulation with residual low-frequency hearing on the ear fitted with the hearing aid. Since we did not set up specific hypotheses about the type of fitting, we regarded the CI recipients as a single group. The age range was 23–83 years (mean 58.2 yrs., sd 16.3 yrs.) for the CI recipients and 27–71 years (mean 60.1 yrs., sd 11.9 yrs.) for the TH participants. The latter did not report any hearing problems and had average pure-tone thresholds of 12.0 ± 7.4 (125 Hz), 9.9 ± 7.7 (250 Hz), 9.7 ± 7.4 (500 Hz), 9.4 ± 5.9 (1 kHz), 12.3 ± 9.3 (2 kHz), 21.1 ± 13.3 (4 kHz), 25.2 ± 17.5 (6 kHz), and 30.4 ± 22.4 dBHL (8 kHz).

All participants gave informed consent to participate in the study before taking part, had normal or corrected-to-normal visual abilities, and were instructed to refrain from caffeine intake at least 6 h prior to the experiment beginning. The study protocol was approved by the local institutional review board (reference 18-257, 30 October 2018).

Table 2 shows the characteristics of the CI recipients.

### 2.6. Data Analyses

Due to the multidimensional approach of data collection, the two experiments generated a relatively large and diverse data set. As a consequence, different statistical methods were used for data analysis. In general, behavioral data (i.e., stress identification, audiovisual gain, reaction times, eye gaze) were analyzed using generalized linear mixed models (GLMMs). In this study, we were particularly interested in the effects of modifying the cues in the different modalities and whether differences could be found between the two study groups. Therefore, modality, modification level, and study group were usually included as fixed factors in the GLMMs. In addition, participants were included as a random factor. In Experiment 2, the congruency condition was considered as an additional fixed factor. The Akaike information criterion (AIC) was used to select the most appropriate model.

A different approach was chosen for the analysis of the task-evoked pupil dilation (TEPD). Pupil data pre-processing and analysis was performed using the CHAP software [45], which included a linear interpolation for blink reconstruction. Following pre-processing, the relative change in pupil dilation from the baseline (interval 500 ms prior to sentence onset) was calculated using the divisive baseline correction equation [45]. TEPD was examined by means of Bayesian temporal analysis with CHAP, applying a paired-sample *t*-test (Cauchy prior width of r = 0.707) to measure effect size, assuming differences between conditions. This analysis had the great advantage, that the time course of the pupil dilation across the entire trial can be considered. Thus, the analysis is not limited to specific time windows, such as the peak pupil dilation or the peak latency, but rather takes into account the morphology of the curves. The Bayes factor BF10 [46] was used to detect differences between conditions by comparing the likelihood of the alternative hypothesis (indicating a difference between two conditions) to the null hypothesis (no differences). For example, a BF10 of 3–10 suggests moderate evidence favoring the alternative hypothesis over the null hypothesis, while a BF10 of 10–30 suggests strong evidence [47].

## 3. Results

Experiment 1: Congruent combination of the single modalities

Experiment 1 was dedicated to the perception of sentence stress in the single modalities and their combination in a congruent audiovisual condition. In line with the research questions and hypotheses outlined in the introduction, the analyses focused on the modification level, modality, and group differences. In contrast, we generally did not distinguish between the different word positions and the virtual characters, since these factors were not the focus of the present study. In principle, however, the two virtual characters yielded similar results, and the stress identification was also similar for the subject and adjective, while it dropped for the object.

- Identification of sentence stress

**Figure 2:**
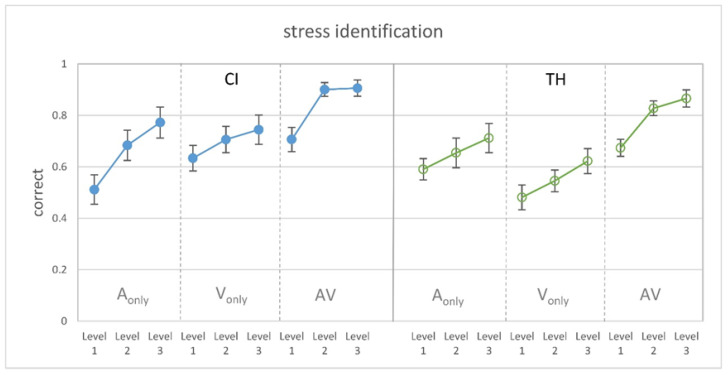
Proportion of correct stress identification for the different modification levels (1 = weak, 2 = intermediate, 3 = strong) and modalities (auditory-only, visual-only, audiovisual). (**Left**): CI recipients. (**Right**): TH participants. Modification levels in the A-only condition consider different changes in F0 for the CI recipients and the TH participants. The graph shows the mean and the standard error of the mean.

Figure 2 shows the proportion of correctly identified stress in the single modalities and in the congruent audiovisual condition related to the three levels of cue manipulation. It can be seen that the modifications had a distinct effect on stress identification for both CI recipients and TH participants, as more salient cues led to a higher performance in the single modalities as well as in the congruent audiovisual condition. As intended by the study design, the presentation of different F0 cues in the auditory-only condition led to similar results for the CI users and TH controls. However, stress identification solely based on visual cues appears to be better for the CI recipients, while the congruent combination of both modalities shows no group differences.

Subjecting the data to a GLMM revealed significant main effects of the modality (F_2,234_ = 43.96, *p* < 0.001) and modification level (F_2,234_ = 18.50, *p* < 0.001), as well as a significant group x modality interaction (F_2,216_ = 3.56, *p* = 0.030). Pairwise comparisons showed that stress identification was significantly better in the AV condition compared to the A-only (T_234_ = 6.33, *p* < 0.001) and the V-only condition (T_234_ = 8.65, *p* < 0.001) and that all modification levels differed significantly from each other (level 1 vs. 2: T_234_ = 4.24, *p* ≤ 0.001; level 1 vs. 3: T_234_ = 6.08, *p* < 0.001; level 2 vs. 3: T_234_ = 2.08, *p* = 0.038). Regarding the group x modality interaction, pairwise comparisons revealed that the study groups did not differ in the AV and A-only modality but that the CI recipients showed a significantly better performance in the V-only condition (T_234_ = 2.73, *p* = 0.007).

**Figure 3:**
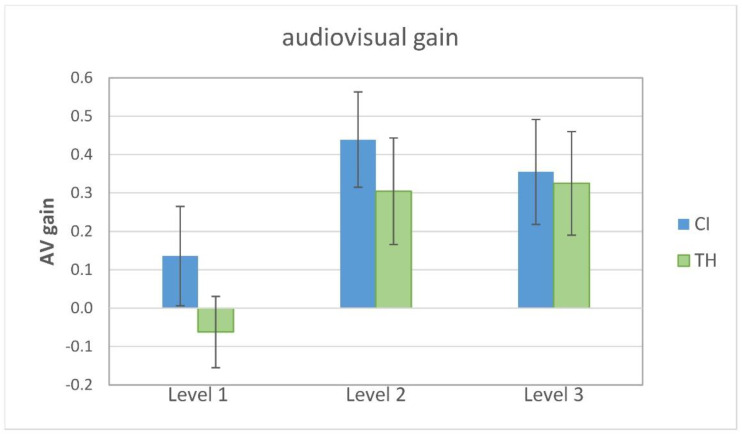
Audiovisual gain for the two study groups related to the modification level (1 = weak, 2 = intermediate, 3 = strong). The graph shows the mean and the standard error of the mean. CI = cochlear implant recipients, TH = typical-hearing participants.

In order to characterize the significantly better performance in the congruent AV condition compared to the unimodal condition, the audiovisual gain (AV gain) was calculated according to Rouger et al. [15], where AV gain = (AV-X)/(1-X) and X is the best unimodal performance. Individually, this can be either the auditory-only or the visual-only condition. Figure 3 shows the AV gain for the two study groups in relation to the modification level. Visual inspection reveals that the CI recipients show a somewhat larger AV gain for all levels, but the pattern appears to be similar for both groups. Specifically, level 1 shows little or no average AV gain.

Subjecting AV gain to a GLMM with the modification level and group as fixed factors and the participant as a random effect revealed a single significant main effect of the modification level (F_2,77_ = 5.75, *p* = 0.005). Pairwise comparisons revealed that the AV gain was significantly smaller for level 1 than for level 2 (T_77_ = 2.95, *p* = 0.013), as well as for level 3 (T_77_ = 2.65, *p* = 0.020). No significant group effect or interaction was found.

- Reaction times

**Figure 4:**
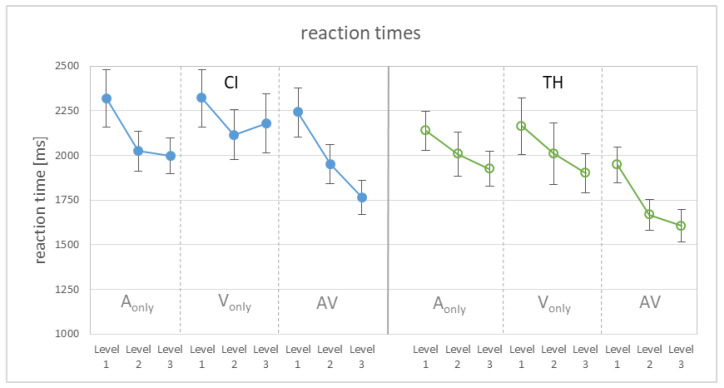
Reaction times relative to the start of the sentence for the different modification levels (1 = weak, 2 = intermediate, 3 = strong) and modalities (auditory-only, visual-only, audiovisual). (**Left**): CI recipients. (**Right**): TH participants. The graphs show the mean and the standard error of the mean.

Reaction times were recorded as a potential behavioral measure of task load, reflecting cognitive demands while processing speech stimuli (e.g., [48,49]). There are basically two options for determining the load: Analyses can be carried out either for the full data set or exclusively for the correct answers, with the latter having the advantage of being independent of performance. However, as both analyses led to qualitatively and quantitatively very similar results, we refer here to the full data set. The average reaction times of the two listener groups for the different modalities and modification levels are shown in Figure 4. The graph again reveals an influence of the modification level and shows the shortest reaction times for the congruent AV condition.

Computing a GLMM on reaction times revealed significant main effects of the modification level (F_2,234_ = 15.45, *p* < 0.001) and modality (F_2,234_ = 18.69, *p* < 0.001). Pairwise comparisons showed that RTs were significantly longer for level 1 than for level 2 (T_234_ = 4.27, *p* < 0.001), as well as for level 3 (T_234_ = 5.40, *p* < 0.001), and longer for the single modalities than the AV condition (T_234_ = 4.72, *p* < 0.001 for A-only, T_234_ = 4.88, *p* < 0.001 for V-only), which themselves did not differ significantly from each other (*p* = 0.44). Though the TH subjects responded on average 171 ms faster than the CI users, the study group did not contribute significantly to the model. Furthermore, none of the interactions were significant.

- Pupillometry

**Figure 5:**
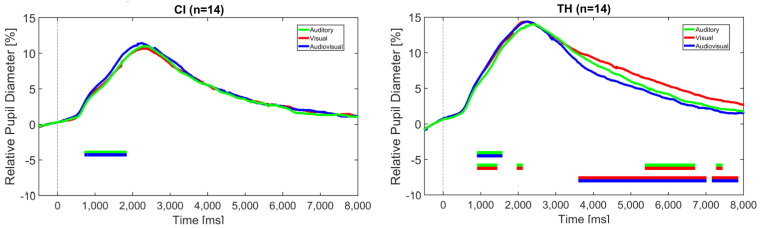
Change in pupil diameter relative to the baseline (interval 500 ms prior to sentence start) over the duration of the trial in the auditory-only, visual-only, and audiovisual modalities. The dashed vertical line indicates the sentence start. The horizontal bars indicate significant differences between the modalities, according to the BF10 factor. (**Left**): CI recipients, (**Right**): TH participants.

Task-evoked pupil dilation (TEPD) was used as a physiological measure of the task load. As with the reaction times, data analysis revealed that TEPD for the entire data set was quantitatively and qualitatively similar to that for correctly identified stress. Since the former is more robust, we opted to use the entire data set regardless of the correctness of the participant’s response. Figure 5 shows the pupil diameter for the two listener groups related to the modality, collapsed across the three modification levels.

Bayesian statistics indicate a significant BF10 for the comparison of the auditory and audiovisual modalities between approximately 1 and 2 s after stimulus onset for the CI recipients. This effect can also be observed in the TH participants, who show additional significant differences between the visual and the audiovisual modality as well as between the visual and the auditory modalities at a later time period at about 4 s until the trial’s end. BF10 at these intervals ranges from 3 to 30, indicating moderate-to-strong evidence for a difference between the conditions—more specifically, identifying sentence stress in the combined audiovisual modality elicits larger pupil dilation than in the auditory modality in both groups between approximately 1 and 2 s. For the TH participants, stress identification in the visual modality results in larger pupil dilation than for the auditory and the audiovisual modalities, suggesting a higher task load in the later period of the trial.

- Eye gaze

**Figure 6:**
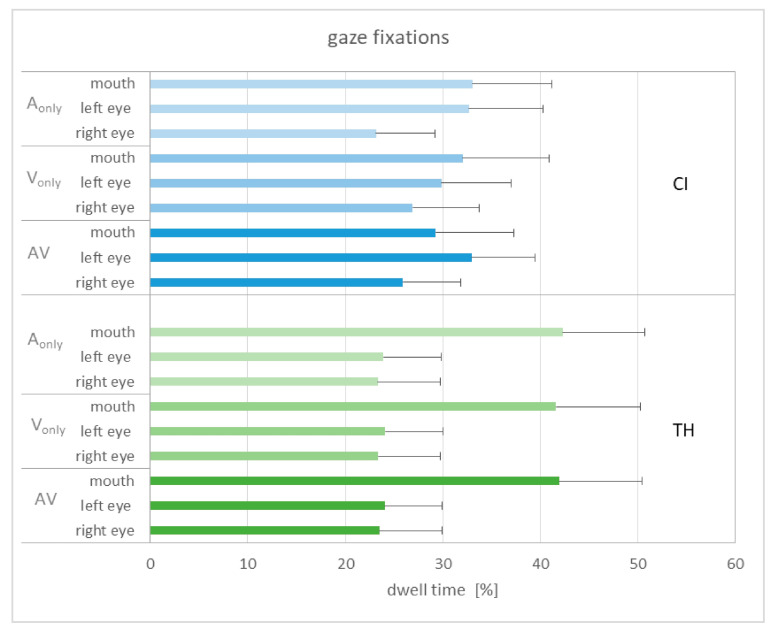
Dwell time related to the areas of interest (left and right eye, mouth) for the different modalities (A, V, AV) and study groups ((**upper**): CI recipients, (**lower**): TH subjects). The mean and the standard error of the mean are shown.

The gaze was considered as an indicator of visual attention and expected to give information on how the participants gained information from facial cues. Three areas of interest were defined, which covered the region of the left and right eyes as well as the mouth (see Figure 1). Figure 6 shows the dwell time for the different areas of interest in relation to the different modalities, collapsed across the three modification levels.

The dwell time does not seem to be related to the modality in general, as very similar patterns can be observed. When comparing the study groups, the TH participants show longer fixations on the mouth, while the CI recipients show equal fixations on the left eye and the mouth. Subjecting the gaze data to a GLMM with the area of interest (AoI), modality, and group as fixed factors and subject as a random factor shows a single significant main effect of AoI (F_2,234_ = 3.45, *p* = 0.034). Pairwise comparisons reveal that the dwell time on the mouth area was significantly longer than on that of the right eye (T_234_ = 2.60, *p* = 0.030). An alternative analysis only separated between fixations on the eyes and the mouth by adding the dwell time of the left and right eyes. This analysis again showed a significant main effect of AoI (F_2,156_ = 9.30, *p* = 0.003). Pairwise comparisons reveal that the dwell time on the eyes was significantly longer than that of the mouth (T_156_ = 3.05, *p* = 0.003). The interaction of AoI and group failed to reach significance (*p* = 0.061), as did the other interactions and main effects.

Experiment 2: Incongruent combination of the single modalities

- Identification of sentence stress

**Figure 7:**
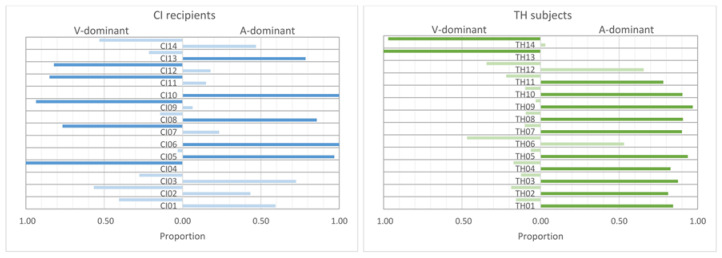
Modality dominance for the responses to the incongruent stimuli. The proportion of answers based on visual or auditory cues, respectively, is shown. Darker colored bars are given if the proportion of responses show a clear (binomial test, *p* ≤ 0.01) dominance of one of the modalities.

Presenting incongruent information to the participants led to the results shown in Figure 7. It displays the proportion of incongruent stimuli with stress identification based on either the visual or the auditory cues. To prevent the participants from giving arbitrary answers when potentially conflicting information was presented, they were not forced to provide a response, as described above. Overall, no responses were given in 7.4% of all trials in the CI recipients and 6.3% in the TH participants. That is, in the vast majority of trials, participants felt able to identify the stressed word in the incongruent stimuli.

Initially, we speculated that modality dominance could depend on cue strength (see Section 2). However, since the stress identification patterns for the incongruent combination were very similar for the natural cue strength and when using the stimuli of modification level 2, we combined the data of these two conditions. In order to identify the possible dominance of the use of one modality over the other, we defined a cut-off value regarding the amount of responses based on the auditory or visual cues. The criterion of the cut-off value was that according to a binomial test one of the modalities was preferred with a *p*-value of 0.01 or less. For example, if 32 responses (see Section 2) were given, this criterion was fulfilled if at least 24 (75%) were determined by one modality. This rather conservative *p*-value was chosen to allow a clear categorization of the participants. The corresponding individuals are indicated by the darker colored bars in Figure 7. On the basis of this criterion, the CI recipients (left panel of Figure 7) showed quite unambiguous patterns in five subjects (CI04, CI07, CI09, CI11, CI12) revealing a clear visual dominance and a clear auditory dominance in another five subjects (CI05, CI06, CI08, CI10, CI13). Using the same criterion, the four remaining subjects could not be assigned to either of these two categories. In contrast, most TH participants (right panel of Figure 7) showed a clear auditory dominance, with the exception of TH06 and TH12. However, two individuals (TH13, TH14) showed visually-dominant behavior.

This pattern corresponds to the stress identification for the congruent stimuli from Experiment 1: Subjecting the stress identification data collected in Experiment 1, as shown in Figure 2, to a GLMM with modality (A-only, V-only, AV), modification level (1, 2, 3), and dominance category (auditory, visual) as fixed factors and subject as a random factor reveals a significant interaction of modality and dominance category (F_4,225_ = 20.10, *p* < 0.001). Pairwise comparisons show that the stress identification was significantly better for the A-dominant group in the auditory domain than for the V-dominant group (T_225_ = 7.57, *p* < 0.001). In contrast, the V-dominant showed better identification in the visual modality. However, this difference did not reach statistical significance (*p* = 0.062).

- Reaction times

To examine the processing of the incongruent multimodal stimuli in more detail, the reaction times were measured and compared with those for the congruent stimuli, which are also presented in Experiment 2. It was found that reaction times were longer in the incongruent condition than in the congruent condition (CI: 4658 ± 652 ms vs. 4340 ± 508 ms, TH: 4250 ± 606 ms vs. 4077 ± 439 ms). Statistical analysis computing a GLMM with study group and congruency as fixed factors and subject as a random factor revealed a single significant main effect of congruency (F_1,51_ = 30.39, *p* < 0.001). The difference between the study groups did not reach significance (*p* = 0.087).

- Pupillometry

**Figure 8:**
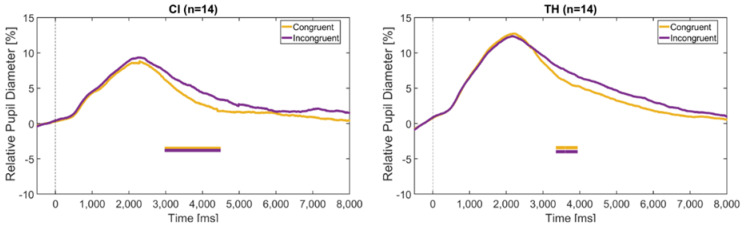
Change in pupil diameter relative to the baseline (interval 500 ms prior to sentence start) over the duration of the trial in the congruent and incongruent condition. The dashed vertical line indicates the sentence onset. The horizontal bars indicate significant differences between the modalities according to the BF10 factor. (**Left**): CI recipients, (**Right**): TH participants.

The time course of the pupil dilation for the congruent and incongruent stimuli is shown in Figure 8. Both the CI recipients and the TH peers show similar curves, with the incongruent stimuli causing a greater change in pupil diameter than the congruent stimuli. This is mainly true for the period starting around 3–4 s after stimulus onset, where BF10 reaches values between 3 and 10.

**Figure 9:**
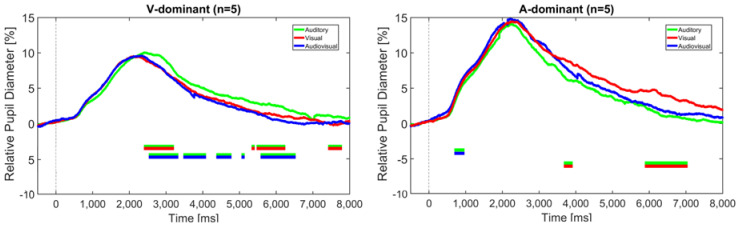
Change in pupil diameter relative to the baseline (interval 500 ms prior to sentence onset) over the duration of the trial in the auditory-only, visual-only, and audiovisual modalities. The dashed vertical line indicates the sentence onset. The horizontal bars indicate significant differences between the modalities according to the BF10 factor. (**Left**): CI recipients with visual-dominant behavior, (**Right**): CI recipients with auditory-dominant behavior.

Since the analysis of sentence stress identification revealed two groups of CI users with opposing modality dominance, as described above, the pupil data from Experiment 1 were reanalyzed accordingly. Figure 9 shows the relative pupil diameter of the visually-dominant group (n = 5, left figure) and the auditory-dominant group (n = 5, right figure) in relation to the different modalities.

It should be noted that these trajectories are based on only five subjects each. However, according to the BF10 factor, significantly greater pupil dilation was found for the auditory modality compared to both the visual and audiovisual modality in the V-dominant group, particularly at later intervals around 3 and 6 s, and for the visual modality compared to the audiovisual modality in the A-dominant group around 6 to 7 s. These differences thus again occurred mainly after the stimulus offset and were also visible when only the correctly identified trials were taken into account. This suggests that the task load was higher in the non-dominant modality.

## 4. Discussion

- Identification of sentence stress

The analysis of stress identification revealed significant main effects of the modification level and the modality as well as a significant modality-by-group interaction. First, the modification level was associated with a significantly different proportion of correct identification—as anticipated. The resulting trajectories were comparable for both study groups, showing that they benefited similarly from the greater salience of the underlying cue. However, with regard to F0, it must be considered that the modifications were much smaller for the TH subjects than the CI recipients, taking into account the differences in F0 perception in CI and TH as quantified by Meister et al. [3,4]. The rationale behind this was, as in Lasfargues-Delannoy et al. [23], to bring the groups to a similar performance level in the auditory modality for the purpose of comparability. Furthermore, a significant main effect of modality was found as the stress identification in the congruent audiovisual condition was better than in the single modalities. Notably, there was a significant group-by-modality interaction as the CI recipients showed better stress identification in the visual-only condition than the TH participants. This superior use of visual cues is consistent with a large body of research demonstrating better abilities in hard-of-hearing individuals in terms of visual speech perception (e.g., [15,16,17]). However, this is in contrast to previous results regarding the perception of linguistic prosody, which showed a poorer use of facial cues in CI recipients for question/statement identification [23]. This finding was unexpected and the authors speculated that the CI users may have been less able to decode and transfer the semantic content of the facial expressions. This ability appears to have been retained by the CI recipients in the present study, as they showed on average roughly 10–20% better stress identification than the TH participants in the V-only condition at all modification levels. It is unclear whether the facial movements for stress identification are more clearly associated with the semantic content of the sentence than the visual cues for distinguishing between questions and statements. Another difference is that Lasfargues-Delannoy et al. [23] used actors who were instructed to reduce their facial movements, whereas we applied controlled stimuli via virtual characters. Thus, the question of whether and how cochlear-implanted patients and typical-hearing individuals differ in the use of visual prosodic cues cannot be answered conclusively.

In order to quantify the benefit of providing congruent multimodal information, we calculated the audiovisual gain relative to the best unimodal condition [15]. Statistical analysis revealed a single significant main effect of the modification level, as level 1 showed the lowest AV gain. A common rationale to explain the beneficial combination of different modalities is the “principle of inverse effectiveness” [50]. Basically, it states that the multimodal gain is largest when perception in the single modalities is poor. Hence, the largest AV gain would have been expected for modification level 1 whereas it should have been small for modification level 3, when the unimodal performance was already quite good. However, in the present study, the AV gain was largest for modification level 2 (mean unimodal performance around 65%), whereas it was smallest for level 1 (mean unimodal performance around 55%). It could therefore be that the cues were too unreliable in the latter case to enable a beneficial combination in the congruent AV condition. Both participant groups showed this pattern, and the CI recipients revealed a somewhat greater gain than the TH participants across all modification levels. However, while Lasfargues-Delannoy et al. [23] showed that their CI patients had a significant three-fold-higher AV gain compared to their normal-hearing peers, the group difference did not reach statistical significance in the present study. Lasfargues-Delannoy et al. also reported that the very small AV gain in their NH controls was not statistically different from zero. The reason for this is unclear. The authors used an eight-channel vocoding system to balance the performance between groups in the auditory modality. It may have been difficult for the NH subjects to combine the unusual vocoded signal with the visual cues provided by the talker’s faces.

Additional insights into the identification of audiovisual sentence stress were provided by the presentation of incongruent stimuli in Experiment 2, which were helpful in identifying individual patterns. According to the theoretical constructs outlined in the introduction [24,25,26], the most reliable modality is used to perform the task. These theoretical considerations are particularly relevant when conflicting information is given in the modalities (such as with incongruent stimuli) and when the perception in one modality is either limited or enhanced (e.g., in the case of the different participant groups). Our initial hypothesis that for typical-hearing individuals auditory cues would show greater reliability and for CI recipients visual cues would show comparably greater reliability predicts different patterns for the two study groups. In fact, as displayed in Figure 7, the TH control group predominantly identified sentence stress based on F0 when given incongruent information, which is consistent with the idea that they use rather auditory than visual cues. However, the group of CI users showed more varied results. Apparently, five subjects each relied more heavily on either the auditory or the visual cues, while the remaining four subjects did not show a clear dominance. That is, contrary to our expectation, CI users may not necessarily use predominantly visual cues for sentence stress identification, but rather show individual behavior. It seems difficult to break down this result to demographic and user-specific data, as outlined in Table 2. For example, one might assume that participants with pre-lingual deafness (CI04) or peri-lingual onset of hearing problems (i.e., CI02, CI03, CI12, CI13, CI14) would more likely belong to the V-dominant group, which is not the case. However, it must be considered that information on both the etiology and onset of hearing loss is often uncertain. Still, as shown with the reanalysis of the Experiment 1 data, modality dominance was linked to the individual ability to use the different cues. This result appears to be generalizable to other contexts. For example, in the classic Mc-Gurk effect, incongruent auditory and visual stimuli are presented, which can result in an integrated perception. In this regard, it was demonstrated that the perception of proficient CI users was primarily based on auditory cues, while non-proficient CI users rather relied on visual cues to solve the conflicting information [51].

- Reaction times

Reaction times (RTs) were assessed as a behavioral measure of task load. RTs were corrected for individually different manual abilities associated with the response scheme (subject—index finger; adjective—middle finger; object—ring finger). As shown in Figure 4 and supported by statistical analysis, RTs were shortest for the most salient modifications in level 3. Thus, the findings from stress identification transferred to reaction times. The same was true when RTs were only considered for the analysis if a correct answer was given. That is, the load decreased as the reliability of the underlying cue increased, as expected.

In addition to the significant contribution of the modification level to the statistical model, the modality also reached significance. In the combined AV condition, RTs were significantly shorter than in the single modalities, which in turn did not differ from each other. The fact that this also applies when only the correct responses were taken into account argues against the idea that this is simply due to the better performance in the AV condition. It is debated whether combining two modalities causes cognitive costs or whether it is rather an automated process (e.g., [30]). While we are not aware of any work addressing this question in the context of multimodal prosody perception, a number of studies have considered audiovisual speech recognition in this regard. For example, Fraser et al. [52] used a dual-task paradigm to show that the AV modality was more effortful than the A-only modality when speech recognition was similar. A paired-associates recall task was used by Picou et al. [29] to assess effort. The rationale behind it is that a higher cognitive load hampers the ability to recall words during a speech recognition task. While speech cognition scores were similar for the AV and A-only condition, variable results were found for the recall task. Specifically, recall in the AV condition was associated with the participant’s lipreading ability, with those having better skills to use visual speech cues showing higher recall—construed as reduced load. Sommers & Phelbs [53] also used a recall task and found that an audiovisual presentation led to better recall than an auditory-only presentation, which was interpreted as less effort required for the former. However, this result only applied to young participants, but not to the group of older individuals, who did not show a modality-dependent difference in recall. Thus, as pointed out by Brown & Strand [30], whether an audiovisual presentation is associated with a higher cognitive load than A-only presentation appears to rely on the method used as well as on various subject-specific factors. With regard to our study, in which a single task reaction time paradigm was used, the results rather suggest a reduction in cognitive load for a congruent audiovisual presentation of sentence stress.

In Experiment 2, it was additionally shown in both study groups that the incongruent multimodal combinations resulted in RTs that were about 200–300 ms longer than the congruent combinations. That is, although most of the participants had a clear dominance of one of the two modalities, the competing information was apparently also processed to a certain degree, presumably increasing the task load. This is in line with Swerts & Krahmer [32], who examined the identification of sentence stress in three different word positions and also found that congruent stimuli yielded faster RTs than incongruent stimuli. This effect was independent of the strong auditory dominance of their normal-hearing participants. Thus, it seems to be difficult to ignore incongruent cross-modal information, despite the dominance according to the modality-specific reliability approaches to the processing of multimodal stimuli [24,25,26].

Furthermore, the results are in line with Föcker et al. [54], who showed that incongruent audiovisual cues of affective prosody contrasting different emotions (happy–angry–sad–neutral) resulted in longer reaction times than congruent combinations. This occurred despite the fact that the participants in their study were instructed to focus on only one modality—either the voice or the face of a talker. Additionally, longer RTs in the incongruent condition could not be explained simply by a response conflict. The authors therefore concluded that the prolonged RTs were the result of interference at early processing stages, before a response selection is made. However, it is unclear to what extent such a conclusion also applies to the results of the present study, since our participants were not instructed to focus on a specific modality.

- Pupil data

The task-evoked pupil dilation (TEPD) was taken as a physiological measure of task load. In general, pupil dilation revealed a typical pattern in both study groups. Compared to the baseline (−500 to 0 ms), the pupil began to dilate at about 500 ms after the stimulus onset (common values: 0.5 to 1.3 s [40]) and reached a plateau after approximately 2.5 s with a mean increase of about 10–15%. In the subsequent trial period, the pupil diameter decreased and re-approached the pre-stimulus value. As mentioned in the Methods Section, TEPD and RTs were assessed in parallel to obtain reaction times in a valid fashion. However, as discussed by Visentin et al. [43], this may have implications for the interpretation of the data, as the pupil reacts to both the presentation of the stimulus and the behavioral response. As a consequence, previous studies have considered a retention phase of several seconds after the presentation of the stimulus and before a response is allowed [40], which, however, is not compatible with measuring valid reaction times. Thus, it can be complicated to separate the pupil dilation caused by the perceptual processing of the stimuli from the participant’s reaction. This is why we interpret our pupil data rather generally as an indication of the task load and not necessarily as the perceptual demand. However, Visentin et al. [43] solved this problem by analyzing only a period of time that corresponded to the listening phase and discarding the subsequent part in which the response was recorded, thus losing potentially interesting information in a later phase of the trial.

In general, different stages can be described in the time course of speech processing, which also applies to prosody perception (e.g., [55,56]). The first, most basic stage involves the sensory (here: uni- and multimodal) analysis of the stimulus. This stage is followed by a subsequent level of phonetic–phonological processing, in which patterns are identified based on the suprasegmental elements. At the next higher level, semantic–syntactic processing commences, in which the patterns are analyzed and finally used at the pragmatic level to understand and interpret the message. When considering the trajectories depicted in Figure 5, Bayesian statistics indicate a significantly higher pupil dilation (BF10 between 3 and 30) for the congruent AV condition compared to the auditory-only modality in the interval between approximately 0.8 to 1.5 s after stimulus onset for both study groups. In addition, there was greater pupil dilation for the visual-only condition compared to the auditory-only condition in the TH participants. Taking into account that the subjects’ mean reaction time was roughly between 1.8 and 2.2 s and that, according to Hupé et al. [57], response preparation can influence pupil dilation 500 ms before the reaction, it is likely that this modality-specific difference stems from the most basic stage described above, namely the sensory–perceptual analysis of the auditory and visual features of the stimulus. This would suggest that the load in the congruent audiovisual condition is higher than in the auditory-only condition, which contrasts the results of the reaction time analysis, as described above. However, it must be noted that RTs do not give any detailed information on the temporal dynamics of the load, as TEPDs can. Furthermore, different measures of load or effort may tap into different domains [58] and thus can lead to different results. An alternative interpretation for the increased pupil dilation in the AV condition is that the participants showed increased arousal once they were aware that both auditory and visual information were available in the stimulus.

Further significant differences (BF10 between 3 and 20) in TEPD were detected later in the trial, at about 4–5 s after the stimulus started until the end at 8 s, but only in the TH participants. Here, the V-only condition led to greater pupil dilation than the A-only condition and the congruent AV combination. This late phase of significant differences cannot be attributed to the sensory–perceptual analysis of the available features, nor to pattern recognition or stress identification at a higher processing level, since responses were already given and, according to Hupé et al. [57], affect pupil size only for a subsequent period of up to 1.5 s. It is, therefore, not clear how this difference in TEPD in the aftermath of stimulus perception and stress identification can be explained. It is possible that the participants may have re-evaluated their response in some way and that this was related to increased load particularly in the V-only modality, which appeared to be the most difficult for the TH participants. A somewhat similar effect was described by Winn et al. [59], who showed a sustained pupil response when the stimulus was too weak to be easily detected. They speculated that their listeners may therefore have drawn on their cognitive resources for a longer period of time.

The fact that no such significant difference was found for the CI recipients may be explained by the different modality dominance, as found in Experiment 2, which vanishes when the grand average across all subjects is considered (see Figure 5). Indeed, reanalyzing the TEPDs by taking the modality dominance into account, as shown in Figure 9, reveals significantly greater pupil dilation in the A-only condition compared to the V-only and AV condition in the visually-dominant group. Vice versa, the auditory-dominant group showed a significantly greater pupil dilation in the V-only condition than in the A-only condition. In both cases, these differences tended to occur in later phases of the trial. Due to the small sample size of only five subjects in each subgroup, these results should be interpreted with caution, however. Still, they are consistent with theories of the reliability-weighted use of cues in the different modalities and are in line with the individual differences in perceptual abilities as described above.

Experiment 2 also showed that incongruent stimuli caused a larger pupil dilation than congruent stimuli (see Figure 8). This is generally in line with the prolonged reaction times shown above and gives further evidence that conflicting information cannot be readily ignored, despite the potential dominance of one of the modalities, and that this increases the task load. This effect was also mainly observed in the advanced phase of the trial, from about 3 to 4 s after stimulus onset, suggesting that it is not necessarily the perceptual processing of incongruent stimuli that is affected, but rather the later stage of decision making and reaction.

- Eye gaze

As described in the Introduction, eyebrow raises give important information about sentence stress and are closely linked to changes in the voice fundamental frequency. We therefore expected that the participants would direct their gaze predominantly on the eye region. Nevertheless, because the mouth shows articulatory movements that are generally important for speech understanding, we included the mouth region as an additional area of interest. In fact, these AoIs covered about 90% of the dwell time, indicating that they account for the vast majority of gaze fixations.

Statistical analysis considered modality, AoI, and study group as fixed factors. It was assumed that gaze fixations in the eye regions would be found particularly for the V-only and the AV condition, but not necessarily for the A-only condition. However, as illustrated in Figure 6 and confirmed by the GLMM, modality had no significant influence on dwell time. This could be due to the stimuli being presented in a mixed-block design that did not prompt expectations about the upcoming information. In other words, the participants were initially unsure whether visual information was present and thus may have explored the stimulus in a similar manner each time.

In contrast, AoI did show a significant effect on dwell time. When considering the left and the right eye separately, the dwell time for the mouth region was significantly higher than for the region of the right eye. Although the fixations on the left eye were stronger than on the right eye, this difference was not statistically significant. However, this is indirectly consistent with Swerts & Krahmer [32], who concluded that the left part of the face provides more salient information about sentence stress than the right part. This conclusion was based on an experiment in which either only the left or the right side of the face was shown. Indeed, stress perception was significantly better when displaying the left part than the right part. The reasons for this finding appear to be inconclusive, and Swerts & Krahmer [32] discuss some possible explanations, such as that movements of the left eyebrow might more strongly correlate with intonation patterns than the right eyebrow.

When only distinguishing between fixations on the eyes or the mouth, we found that the dwell time on the eye regions was significantly larger than on the mouth region, also in line with the results of Swerts & Krahmer [32] that the upper part of the face is more important for sentence stress perception than the lower part. Nevertheless, a relatively large proportion of the fixations were actually directed at the mouth, although there were only articulatory movements that could contribute to understanding the sentence but did not provide reliable information about the stressed word.

Contrary to our expectation, we did not find any significant interaction for the dwell time between the study group and the AoI in the present study. This stands in contrast to Lasfargues-Delannoy et al. [23], who found more gaze fixations on the mouth than on the eyes for their CI recipients compared to normal-hearing listeners. It may be speculated that CI recipients are more accustomed to extracting speech information from articulatory movements and that this behavior persists even when speech cues from the mouth are irrelevant for the task. In contrast, in the present study this effect appeared to be more pronounced in the TH subjects, as demonstrated in Figure 6, although the group by AoI interaction did not reach significance. However, when Lasfargues-Delannoy et al. [23] divided their NH listeners into young and older subjects, they found that the gaze fixations of the CI users were similar to those of the latter, pointing to an age-related effect.

Although the gaze provides valuable information about the participants’ visual attention, it should not be seen as an ultimate measure since peripheral vision can also play a role. Peripheral vision refers to the fact that an area beyond the point of fixation is also perceived and is important for many real-world tasks (overview in [60]). Since the visual angles in our experiment were relatively small, as described above, it is likely that facial movements outside the fixation point were also detected. Furthermore, there is some evidence that this effect is more pronounced in individuals with hearing loss [61,62], a factor that may have played a role in the comparison between our CI recipients and TH subjects.

### Strengths and Limitations of the Study and Future Directions

The aim of the present study was to provide a detailed description of multimodal sentence stress perception in CI recipients in comparison to TH peers by applying fine-grained levels of underlying cue strength. To this end, virtual characters were used that allowed highly controlled and highly realistic stimuli. Another strength of the study is that it considers various aspects of sentence stress perception, covering identification, reaction times, task-evoked pupil dilation, and gaze fixation, thus potentially providing more detailed insights into the underlying mechanisms.

However, despite the advantages for the purpose of this study, the use of virtual characters may also impose some limitations. Even though the video-realistic appearance and the very high similarity of movements compared to the captured real talker (see [39]), virtual characters may not cover all details of facial expressions that are present in a real scenario. Nevertheless, we claim that the most important cues are provided in a reliable manner. Moreover, using only two virtual characters may limit the generalizability of the findings, as visual stress cues can differ individually [8]. Generalizability may also be limited by using only one sentence, although this choice was beneficial for controlling the cues of interest [32].

One area for future research is the extent to which user- and device-specific factors may influence the results. For example, it can be assumed that post-lingually deafened individuals have better access to acoustic prosody cues. The same assumption applies to patients who communicate in the oral mode. In addition, there may be differences depending on the fitting, as residual acoustic hearing may provide better access to changes in the voice fundamental frequency in bimodally fitted patients. Indeed, visual inspection of the data revealed that the latter showed somewhat better stress identification in the auditory-only modality than the participants who were uni- or bilaterally fitted. However, our sample was not suitable to address these aspects in a thorough manner. Another factor that might have played a role in stress identification for the visual modality may be the speech-reading ability of the participants, though no stress cues were given by articulatory movements of the virtual characters’ mouth. Nevertheless, the potential influence of lipreading on the results should be considered in future examinations.

## 5. Conclusions

This study shows that cochlear implant users had a significantly better ability to identify the stressed word based on facial cues than their typical-hearing peers. However, the two study groups benefited from the congruent combination of auditory and visual cues in a similar manner. The effect of combining the two modalities was highly significant and relatively large, with about 15–20% better stress identification compared to the unimodal presentation. Task load as assessed by the reaction times was significantly lower in the AV condition compared to the unimodal conditions. Task-evoked pupil dilation as a physiological measure of task load revealed higher pupil dilation during the presentation of combined audiovisual cues in both groups and greater pupil dilation for the visual modality compared to the auditory modality in the TH subjects, suggesting that the two load measures may tap into different domains. Incongruent combinations of the auditory and visual cues revealed that the majority of the TH subjects identified the stressed word based on changes in F0, while the CI recipients showed more individual patterns with some relying more on auditory cues and some relying more on visual cues.

These findings may also provide valuable information in a clinical context. While the assessment of speech recognition is an important clinical focus, other vital factors of speech perception, such as prosody, have hardly been taken into account to date. However, it is not necessarily possible to draw conclusions from speech audiometric data on the perception of prosody in general or sentence stress in particular. Therefore, multimodal testing and possibly considering training of these aspects would take more account of individual speech perception in everyday life than speech audiometry alone.

## Figures and Tables

**Table 1:** Stimulus parameters for the three different modification levels (1 = weak, 2 = intermediate, 3 = strong) for the audio-only manipulations (F0/Hz), the visual-only manipulations (expression strength/%), and the congruent audiovisual combination. CI = cochlear implant users, TH = typical-hearing participants.

	Audio-Only			Visual-Only			Audiovisual		
**Modification** **level**	1	2	3	1	2	3	1	2	3
**CI**	10 Hz	20 Hz	40 Hz	10%	30%	50%	10 Hz 10%	20 Hz 30%	40 Hz 50%
**TH**	6 Hz	8 Hz	10 Hz	10%	30%	50%	6 Hz 10%	8 Hz 30%	10 Hz 50%

**Table 2:** Characteristics of the CI recipients.

ID	Age [yrs.]	Gender	Etiology of Hearing Loss	Device Left	Device Right	CI Use Since	HA Use Since
CI01	65	Male	Sudden hearing loss	----	CI (CP910, Cochlear Ltd., Sydney, Australia)	2003	----
CI02	67	Female	Unknown	HA (Enzo Q, GN ReSound, Ballerup, Denmark)	CI (CP1000, Cochlear Ltd., Sydney, Australia)	2018	1963
CI03	29	Male	Unknown	HA (type unknown, Phonak, Stäfa, Switzerland)	CI (CP1000, Cochlear Ltd., Sydney, Australia)	2014	1999
CI04	23	Male	Pneumococcal meningitis at 7 months	CI (CP1000, Cochlear Ltd., Sydney, Australia)	CI (CP1000, Cochlear Ltd., Sydney, Australia)	2001	----
CI05	38	Male	Progressive hearing loss	CI (CP1000, Cochlear Ltd., Sydney, Australia)	CI (CP1000, Cochlear Ltd., Sydney, Australia)	2021	2019
CI06	60	Male	Genetic	CI (Sonnet-2, MED-EL, Innsbruck, Austria)	CI (Sonnet-2, MED-EL, Innsbruck, Austria)	2013	1982
CI07	51	Female	Genetic	HA (type unknown, Phonak, Stäfa, Switzerland)	CI (CP1000, Cochlear Ltd., Sydney, Australia)	2017	1998
CI08	83	Female	Presbycusis	CI (CP1000, Cochlear Ltd., Sydney, Australia)	HA (Audeo yess III, Phonak, Stäfa, Switzerland)	2004	1959
CI09	74	Female	Presbycusis	CI (CP1000, Cochlear Ltd., Sydney, Australia)	CI (CP1000, Cochlear Ltd., Sydney, Australia)	2015	1985
CI10	68	Male	Sudden hearing loss	CI (Naida CI Q90, Advanced Bionics LLC, Valencia, CA, USA)	HA (B 70 Naida UP, Phonak, Stäfa, Switzerland)	2017	1997
CI11	65	Male	Right: unknown, left: labyrinthine aplasia	HA (Signia Pure 3X P312, Sivantos Inc., Piscataway, NJ, USA)	CI (CP1000, Cochlear Ltd., Sydney, Australia)	2018	1992
CI12	65	Male	Severe middle ear infection at 6 years	CI (Sonnet2, MED-EL, Innsbruck, Austria)	CI (Sonnet2, MED-EL, Innsbruck, Austria)	2018	2000
CI13	68	Female	Unknown	CI (Sonnet1, MED-EL, Innsbruck, Austria)	CI (Sonnet1, MED-EL, Innsbruck, Austria)	2017	1961
CI14	57	Female	Recurring middle ear infections in childhood	HA (ENJOY E-FA 50, Widex, Lynge, Denmark)	CI (CP1110, Cochlear Ltd., Sydney, Australia)	2023	2008

## Data Availability

The data presented in this study are available upon reasonable request from the corresponding author.
